# Hereditary hypophosphatemia in Norway: a retrospective population-based study of genotypes, phenotypes, and treatment complications

**DOI:** 10.1530/EJE-15-0515

**Published:** 2015-02

**Authors:** Silje Rafaelsen, Stefan Johansson, Helge Ræder, Robert Bjerknes

**Affiliations:** 1 Section for Pediatrics, Department of Clinical Science, Haukeland University Hospital, University of Bergen, N-5021, Bergen, Norway; 2 Center for Medical Genetics and Molecular Medicine, Haukeland University Hospital, Bergen, Norway; 3 Department of Pediatrics, Haukeland University Hospital, Bergen, Norway

## Abstract

**Objective:**

Hereditary hypophosphatemias (HH) are rare monogenic conditions characterized by decreased renal tubular phosphate reabsorption. The aim of this study was to explore the prevalence, genotypes, phenotypic spectrum, treatment response, and complications of treatment in the Norwegian population of children with HH.

**Design:**

Retrospective national cohort study.

**Methods:**

Sanger sequencing and multiplex ligand-dependent probe amplification analysis of *PHEX* and Sanger sequencing of *FGF23*, *DMP1*, *ENPP1KL*, and *FAM20C* were performed to assess genotype in patients with HH with or without rickets in all pediatric hospital departments across Norway. Patients with hypercalcuria were screened for *SLC34A3* mutations. In one family, exome sequencing was performed. Information from the patients' medical records was collected for the evaluation of phenotype.

**Results:**

Twety-eight patients with HH (18 females and ten males) from 19 different families were identified. X-linked dominant hypophosphatemic rickets (XLHR) was confirmed in 21 children from 13 families. The total number of inhabitants in Norway aged 18 or below by 1st January 2010 was 1 109 156, giving an XLHR prevalence of ∼1 in 60 000 Norwegian children. *FAM20C* mutations were found in two brothers and *SLC34A3* mutations in one patient. In XLHR, growth was compromised in spite of treatment with oral phosphate and active vitamin D compounds, with males tending to be more affected than females. Nephrocalcinosis tended to be slightly more common in patients starting treatment before 1 year of age, and was associated with higher average treatment doses of phosphate. However, none of these differences reached statistical significance.

**Conclusions:**

We present the first national cohort of HH in children. The prevalence of XLHR seems to be lower in Norwegian children than reported earlier.

## Introduction

Hereditary hypophosphatemia (HH) is a group of rare diseases with disordered phosphate metabolism and decreased renal tubular phosphate reabsorption (1). In hypophosphatemic rickets (HR), the hypophosphatemia is associated with rickets and osteomalacia, whereas syndromes with hypophosphatemia combined with osteosclerosis and ectopic calcifications, and not rickets or osteomalacia, are also recognized (1).

HR can be classified as either dependent or independent of the bone derived fibroblast growth factor 23 (FGF23) (1). FGF23 is a phosphate-regulating hormone (2), acting on kidney tubuli cells to decrease expression of sodium-phosphate co-transporter types IIa and IIc (NaPi-IIa and NaPi-IIc) encoded by *SLC34A1* and *SLC34A3* respectively. Elevated levels of serum phosphate increase the expression of FGF23 thereby decreasing the reabsorption of phosphate in the renal proximal tubule, while hypophosphatemia normally down regulates the expression of FGF23. FGF23 also down regulates the1-α-hydroxylase (encoded by *CYP27b1*), thus inhibiting the activation of 25OH vitamin D (25OHD) to 1,25(OH)_2_ vitamin D (1,25(OH)_2_D), and up regulates 24-hydroxylase (encoded by *CYP24a1*), which inactivates 1,25(OH)_2_D by conversion to 24,25(OH)_2_ vitamin D (3). In FGF23-dependent HR, the physiological increase in serum 1,25(OH)_2_D in response to hypophosphatemia is blunted, and the result is a serum level of 1,25(OH)_2_D that is low, or inappropriately normal for the degree of hypophosphatemia (4).

FGF23 dependent HR is caused by mutations in genes involved in the FGF23 bone–kidney-axis, with levels of intact FGF23 (iFGF23) being elevated or inappropriately normal in the setting of hypophosphatemia when suppressed FGF23 is to be expected (1). FGF23 dependent HR includes X-linked dominant HR (XLHR) caused by loss-of-function mutations in the phosphate regulating endopeptidase homolog, X-linked (*PHEX*) gene (5), autosomal dominant HR caused by gain of function mutations in the *FGF23* gene (6), and three types of autosomal recessive HR. ARHR1 is caused by mutations in the *DMP1* gene, encoding the dentin matrix protein 1 (7, 8), ARHR2 is caused by mutations in the *ENPP1* gene encoding the ectonucleotide pyrophosphatase/phosphodiesterase 1 (9, 10), whereas we have recently shown an association between biallelic mutations in *FAM20C* and FGF23-dependent ARHR3 in a Norwegian family (11). FAM20C encodes a protein kinase, important in many phosphorylation processes. Phosphorylation of FGF23 by FAM20C makes FGF23 less stable by inhibiting *O*-glycosylation by GalNacT3 (12), and inactivating mutations in FAM20C thus leads to increased levels of iFGF23 (11, 13). There is also one report of FGF23 dependent HR caused by an activating translocation leading to up-regulation of the expression of the KL gene, encoding the anti-aging protein α-klotho (14). In FGF23-independent HR, as seen in hereditary HR with hypercalcuria (HHRH) caused by mutations in the *SLC34A3* gene (15, 16), the level of iFGF23 is appropriately down-regulated (16).

Treatment of HR includes oral phosphate replacement several times daily, combined with calcitriol to counteract the secondary hyperparathyroidism (HPT) elicited by the serum phosphate peak (17) and transient decrease in serum ionized calcium upon phosphate dosing. Treatment is balanced to improve linear growth and reduce skeletal deformities while simultaneously minimizing the risk of complications to treatment such as secondary and tertiary HPT, nephrocalcinosis, hypertension, and renal failure (18).

We have conducted the first complete national study of HH in children, to explore the prevalence, genotypes, phenotypic spectrum, and response to and complications of treatment.

## Subjects and methods

### Patient population

During 2009 all pediatric hospital departments in Norway were contacted to identify children with HH. The number of patients identified was compared to the number of patients younger than 18 years registered in the Norwegian Patient Registry (NPR) with the diagnosis code ‘E83.3 Disorders of phosphorus metabolism and phosphatases’ in the World Health Organization's International Classification of Diseases version 10 (WHO ICD-10). Patients were continuously recruited through the years 2009–2014.

The inclusion criteria for HH were serum phosphate below the age dependent reference range in repeated samples combined with tubular maximum reabsorption rate of phosphate per glomerular filtration rate (TmP/GFR) not due to primary HPT, HPT secondary to renal failure or malabsorption, Fanconi syndrome or other tubulopathy, vitamin D dependent rickets, vitamin D deficiency or hypophosphatemia secondary to acute metabolic derangements. A family history or genetic diagnosis was supportive, but not required for inclusion.

### Genetic analysis

Genomic DNA was purified from blood using the QiaSymphony System (Qiagen). If the mutation status was not already known, all exons and intron–exon boundaries of *PHEX* were sequenced in the index case of each family. If a disease causing mutation was not found, and the inheritance pattern suggested a sporadic case or X-linked dominant disease, multiplex ligand-dependent probe amplification (MLPA) analysis of *PHEX* were performed at the Molecular Genetics Laboratory, Royal Devon and Exeter Foundation NHS Trust, Exeter, Devon, UK. The *PHEX* MLPA analysis can identify mid-size deletions and insertions not detected by regular Sanger sequencing or chromosomal analysis.

All exons and intron–exon boundaries of *FGF23*, *DMP1*, *ENPP1*, *KL*, and *FAM20C* were sequenced, in successive order, in subjects without pathogenic *PHEX* mutations.

In short, DNA targets were first amplified by PCR (list of primers available upon request) using the AmpliTaq Gold DNA Polymerase System (Applied Biosystems). PCR amplicons were purified with 2 μl of ExoSapIT. Using the Big Dye Terminator chemistry sequencing was performed on the 3730 DNA analyzer (Applied Biosystems) and analyzed using the SeqScape Software (Applied Biosystems).

All mutations detected were compared to variants previously reported in the SNP database (http://www.ncbi.nlm.nih.gov/projects/SNP/index.html) and in the PHEX database (http://www.pahdb.mcgill.ca/cgi-bin/phexdb/phexdb_mutQ1.cgi?field=ID_mut&value=).

### Review of medical history

Information on age at diagnosis, clinical and biochemical findings at diagnosis, treatment, and complications was collected by review of the medical records of included patients. Height was converted to *z*-scores according to Norwegian growth charts (19). Delta *z*-score was calculated as the difference between *z*-score at last registered consultation and *z*-score at diagnosis. Laboratory data from each visit from the time of diagnosis to the time of inclusion in the study, including serum levels of calcium, phosphate, alkaline phosphatase, creatinine, parathyroid hormone (PTH), 25OHD, and 1,25(OH)_2_D were also recorded, as well as results from kidney ultrasound and skeletal X-ray examinations. TmP/GFR was calculated according to the formula provided by Barth *et al*. (20). Blood tests were analyzed according to each hospital laboratory's current standard methods.

### Genotype–phenotype associations in XLHR patients

The *PHEX* mutations were classified as either deleterious or plausible according to earlier studies (21). Deleterious mutations comprise those leading to a premature stop codon, including nonsense mutations, splice-site mutations, and insertions and deletions affecting reading frame. Mutations classified as plausible were missense mutations and deletions that did not affect reading frame. The phenotypic features compared were age at diagnosis and at the last registered consultation, height *z*-score at diagnosis and at the last registered consultation, serum levels of phosphate, ALP and PTH at diagnosis, skeletal manifestations (clinical or radiological signs of rickets or bowing) at diagnosis, and information on dental involvement, nephrocalcinosis, and persistent bowing at the last registered consultation.

### Statistical analysis

The prevalences of HH and XLHR was calculated based on the number of patients aged 0–18 years registered with these diagnosis in 2009 and the total number of people in Norway aged 0–18 years by 1st January 2010, obtained from the official Statistics Norway database (22).

The data were analyzed with SPSS version 22. Between-group comparisons were performed using non-parametric tests; medians were compared using the Mann–Whitney *U* test, and frequencies were compared with the Fisher's exact test.

### Ethics and approvals

Written informed consent was obtained from all study participants. The study was approved by the Regional Committee for Medical and Health Research Ethics, Region West, Norway (REK number 2009/1140).

Clinical Trial Registration (ClinicalTrials.gov) number: NCT01057186.

## Results

### HH patient cohort

By 31st December 2009 we had identified a total of 23 children aged 0–18 years with HH in Norway, and all except one were included in this study. Two additional patients with HH, one with confirmed XLHR, were born before 2009, but diagnosed after 2010. By the end of 2009 the National Patient Registry reported 32 children with the ICD-10 diagnosis ‘E83.3 Disorders of phosphate metabolism and phosphatases’, but four of these patients had hypophosphatasia, and five had transient hypophosphatemia in the course of malignancy, premature birth, or other underlying condition. On 1st January 2010, the number of inhabitants aged below 18 years was 1 109 156, and this gives a prevalence of HH of ∼1 in 45 000 children. XLHR was confirmed in 18 children, giving a prevalence of ∼1 in 60 000. During the period from 1st January 2010 to 31st December 2014, we included another four patients, two of which immigrated to Norway in 2014 and two patients born to XLHR mothers after 2010.

The total of 28 patients included comprised 18 females and ten males from 19 different families (Supplementary Figure 1, see section on supplementary data given at the end of this article). XLHR was confirmed in 21 children. Twenty-two patients had a family history of HR, while six were sporadic cases.

### Genotypes in HH

We identified the likely pathogenic mutation in 15 of the 19 HH pedigrees (79%). *PHEX* mutations were found in 21 subjects from 13 different pedigrees (Supplementary Table 1, see section on supplementary data given at the end of this article), and three of the XLHR probands were sporadic. Of the 13 different *PHEX* mutations detected, nine had not been previously reported in the SNP or PHEX databases (see section ‘Materials and methods’). The nine novel mutations comprised one large duplication, two single nucleotide deletions leading to frameshift and premature stop codons, two triplet deletions leading to loss of one or more codons, two missense mutations, one nonsense mutation, and one splice site mutation. One male patient with HHRH was found to be compound heterozygous for a splicing mutation, c.757-1G>A, and an intronic deletion mutation, c.925+20_926-48del, in the *SLC34A3* gene. The c.757-1G>A affects the conserved splice donor site of intron 7, and is predicted to cause aberrant splicing. The c.925+20_926-48del mutation has been reported previously (15). Two patients with combined heterozygous mutations in *FAM20C* are described elsewhere (11). In four patients, two sporadic cases in females and two males with affected mothers, we were not able to identify a pathogenic mutation by standard Sanger sequencing of *PHEX*, *FGF23*, *DMP1*, *ENPP1* or *KL*, or by *PHEX* MLPA.

### Phenotypes in HH

The median age at diagnosis was 2.1 years (range 0.1–15.5 years), and 26 of the 28 subjects were diagnosed before the age of 7 years (Table 1 and detailed information for each subject is given in Supplementary Table 2, see section on supplementary data given at the end of this article). Median age at the last registered consultation was 12.1 years (range 1.3–18.3).

#### Phenotype in XLHR

**Figure 1:**
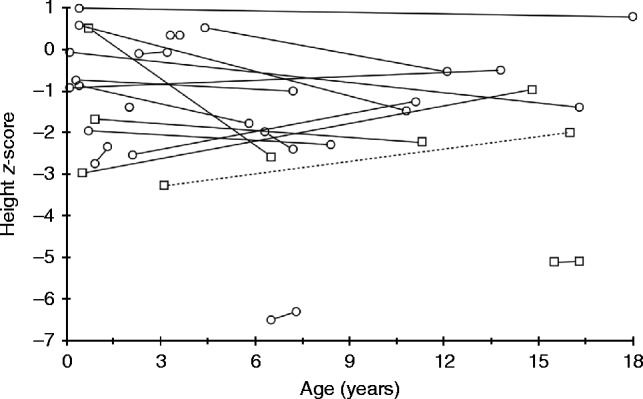
Growth in X-linked hypophosphatemic rickets. Ages at diagnosis and last registered consultation, and the corresponding height *z*-scores for 19 of the 21 XLHR patients. The two outliers represent two immigrant siblings who had not received any medical care and did not start treatment until age 6 and 15 years respectively. The broken line represents the male treated with growth hormone. Circles represent females and squares represent males.

The 21 XLHR children comprised 16 females and five males. Their median age was 0.9 years (range 0.1–15.5) at diagnosis, and 10.8 years (range 1.3–18.0) at the last registered consultation. Growth was compromised, and Fig. 1 illustrates the height *z*-scores for 19 of the 21 XLHR patients related to age at diagnosis and at the last registered consultation. Males tended to have a lower height *z*-score than females (Table 2), both at diagnosis and at the last registered consultation, whereas delta *z*-score did not differ between the sexes. In accordance with an earlier study (23), we analyzed the XLHR patients' data depending on initiation of treatment before or after 1 year of age. There was no significant improvement in height *z*-score in either treatment group. One patient was treated with growth hormone (GH) from the age of 11 years 10 months. His height *z*-score improved from −2.9 at the last consultation before initiation of GH to a final height of −1.9 s.d. at age 17 years (data not shown).

Clinical or radiological evidence of skeletal involvement was found in 13 of 20 children (four out of five males and nine out of 15 females) at diagnosis. The seven patients without skeletal manifestations at diagnosis were all familial cases, diagnosed before the age of 8 months (median 4 months), and comprised six females and one male. During the years after diagnosis, all of these had episodes of rickets identified on clinical or radiological examination, and a male and two of the females had persisting skeletal axis deviations at the last registered consultation. Overall, nine females and four males had persisting axis deviation at the last registered consultation, and correcting osteotomy had been performed in one female and two males. The prevalence of dental involvement was higher in male than female XLHR patients, and in children who started treatment after the age of 1 year (Table 2).

#### Genotype–phenotype associations in XLHR

There were no differences between the mutation status groups in growth, dental involvement, persistent bowing, or development of nephrocalcinosis (results not shown).

### Treatment and complications in HH

The median age at the start of treatment was 2.1 years. Twenty-six of the 28 patients were treated with oral phosphate and vitamin D (alfacalcidol) supplements (Table 1). Two patients were diagnosed at the time of inclusion, and had not started any treatment at that point.

#### Treatment and complications in XLHR

Details of medical treatment were available for 19 of the 21 XLHR patients. In this group, the median age at the start of treatment with oral phosphate and alfacalcidol was 1.0 year (range 0.2–15.6), and ten of 19 children started treatment before the age of 1 year.

Information concerning development of nephrocalcinosis was available for 20 of 21 XLHR patients, and nephrocalcinosis was diagnosed in nine of 20 (45%), at a median age 4 years 6 months (range 1 year–5 years 5 months), after a median time in treatment of 1 year 5 months (range 8 months–4 years 5 months). The median time in treatment for patients without registered nephrocalcinosis was 7 years 2 months (range 0–14 years 7 months).

**Figure 2:**
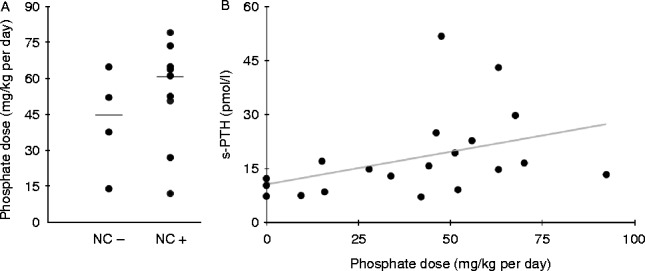
Complications in X-linked hypophosphatemic rickets. (A) Nephrocalcinosis: the average daily phosphate (given as mg/kg per day elemental phosphorus) dose in patients who developed nephrocalcinosis (NC+) and patients who did not (NC−). The horizontal lines represent the median in each group. (B) Hyperparathyroidism: the relationship between the maximum registered value of serum PTH and phosphate dose (given as mg/kg per day elemental phosphorus) at the same time point.

All nine XLHR patients who developed nephrocalcinosis did so within 5 years of treatment. Of the 11 patients without nephrocalcinosis, only four had been treated for 5 years or more, and were included in further comparisons. The prevalence of nephrocalcinosis in this subgroup was nine of 13 (69%). There was a trend toward a higher average daily dose of phosphate (given as mg/kg per day elemental phosphorus) during the years before the diagnosis of nephrocalcinosis as compared to the daily phosphate dose during the first 5 treatment years in patients without nephrocalcinosis (Fig. 2A) (median 61.0 mg/kg per day (range 12.1–79.0) and median 44.8 mg/kg per day (range 13.8–64.7) respectively). Moreover, there was a tendency for earlier start of treatment in children who developed nephrocalcinosis compared with children that did not (median 0.5 year vs 1 year; range 0.2–4.4 vs 0.6–3.6), and seven of nine children with nephrocalcinosis and two of four children without nephrocalcinosis had started treatment before 1 year of age. There were no differences in the starting doses of phosphate and alfacalcidol, average daily dose of alfacalcidol, serum level of PTH level at diagnosis, maximum registered serum PTH, or maximum registered urine-calcium/creatinine ratio (U-Ca/creatinine; results not shown). Furthermore, the groups did not differ with respect to the occurrence of skeletal symptoms at diagnosis, dental involvement at diagnosis, persistent bowing at the last registered consultation, or delta height *z*-score (not shown).

Information concerning parathyroid state was available in 18 patients, of whom 16 had elevated levels of total intact PTH at the time of diagnosis (Table 1 and Supplementary Table 2a). All patients developed transient HPT during treatment in the face of normocalcemia. As seen in Fig. 2B, there was a positive association between the maximum measured serum PTH and the daily dose of phosphate (given as mg/kg per day of elemental phosphorus). Tertiary HPT was diagnosed in one female XLHR patient at 13 years of age. She had been treated with phosphate and alfacalcidol from the age of 5 months, and during the 12.5 years of treatment, the average phosphate dose was 83.0 mg/kg per day (range 47.0–127.0 mg/kg per day) and alfacalcidol dose 18.5 ng/kg per day (range 11.4–44.0 ng/kg per day). Treatment with calcimimetics was started, and she has avoided the need of parathyroidectomy (24).

#### Treatment and complications in non-X-linked HH

Nephrocalcinosis was diagnosed in one female patient with no detected mutation in any of the known genes at age 8 years 2 months after 6 years 4 months of treatment with phosphate and alfacalcidol. Nephrocalcinosis was also demonstrated in the male patient with HHRH, before start of treatment. Tertiary HPT was found in one female patient with no established mutations in any of the known genes. She had been treated for 14 years, with an average dose of elemental phosphorus of 45.9 mg/kg per day (range 38–80 mg/kg per day) and alfacalcidol 34.2 ng/kg per day (range 22–49.6 ng/kg per day) the last 7 years before the development of permanently elevated PTH combined with hypercalcemia. The patient has responded well to treatment with a calcimimetic, and has so far not needed parathyroidectomy.

## Discussion

We have presented the first national cohort of HH and XLHR in children, describing the prevalence, genotypes, phenotypic spectrum, and response to and complications of treatment in the Norwegian pediatric population. The prevalence of XLHR in Norwegian children was one in 60 000. Earlier reports from regional cohorts, with a risk of selection bias, have found the prevalence of XLHR to be ∼1 in 20 000 (25, 26). Studies of large pedigrees of XLHR patients have reported a low penetrance of skeletal manifestations in hypophosphatemic female family members, whereas all hypophosphatemic males had skeletal manifestations of disease (27). Hence, there is a possibility of undiagnosed XLHR in Norwegian females from pedigrees without affected males. However, the ratio of female to male patients in our cohort was 16:5, as compared to the expected ratio of 2:1 for X-linked dominant disorders; a large proportion of undiagnosed females thus seems unlikely. Since our study included only children already in contact with health care and asymptomatic members of the pedigrees were not tested for hypophosphatemia, we cannot rule out hypophosphatemic second-degree relatives (28). It is therefore possible that the prevalence of HH and XLHR in the Norwegian pediatric population may be higher than one in 45 000 and one in 60 000 respectively.

We identified the genotype responsible for HH in 79% of pedigrees in this population-based cohort, and *PHEX* mutations comprised 87% of the verified mutations. This supports what has been found by others (29), and confirms that XLHR is the most common variant of HR. Of 13 *PHEX* mutations, nine (69%) had not been reported earlier (ExAC Browser accessed 21.05.15, http://exac.broadinstitute.org/gene/ENSG00000102174), demonstrating that most mutations are private in this gene (28). We have previously reported two male siblings with the first identified association between compound heterozygous mutations in *FAM20C* and FGF23 dependent hypophosphatemia in humans (11). None of the patients had mutations in *FGF23*, *DMP1*, *ENPP1*, or *KL*, confirming that mutations in these genes rarely seem to be the cause of HH. In four patients we did not find the likely disease causing mutation. However, as illustrated by our finding of *FAM20C* mutations (11), there are possibilities of mutations in other genes associated with pathways involving FGF23, phosphate reabsorption, and tissue mineralization.

One adolescent male was compound heterozygous for mutations in the *SLC34A3* gene. He had no manifestations of rickets, normal growth and bone mineral density, and came to medical attention because of recurrent kidney stones, accompanied by hypercalcuria, hypophosphatemia, suppressed PTH, and high 1,25(OH)_2_D. He had a novel splicing mutation c.757-1G>A affecting the conserved splice donor site of intron 7, predicted to cause aberrant splicing, and a previously reported intronic deletion mutation, c.925+20_926-48del (15). Earlier studies have shown that about 10% of homozygous and 16% of compound heterozygous carriers of mutations in *SLC34A3* presented with renal calcifications, without evidence of skeletal involvement (30, 31). Thus, our case is consistent with a phenotypic and genotypic heterogenesity in *SLC34A3* related conditions, including HHRH.

When comparing non-sense *PHEX* mutations with missense *PHEX* mutations likely to reduce protein function, we did not find differences in growth, severity of skeletal or dental disease, or in the prevalence of treatment complications based on the type of mutation. Our findings confirm the results of another recent study (21), whereas other studies have suggested an association between truncating mutations and a more severe skeletal phenotype (32, 33, 34). However, even in subjects with the same genotype, the skeletal phenotype seems to be very variable and individual (35, 36). This might reflect influence from other genetic variants in mineralization and phosphate metabolism. Interestingly, it was recently reported that patients homozygous or heterozygous for the *FGF23* sequence variant c.C716T (p.T239M, rs7955866) had significantly lower levels of serum phosphate and lower renal TmP/GFR than patients homozygous for the WT allele (37). Another research group have reported a weak, but significant association between the c.C716T variant of *FGF23* and lower TmP/GFR and lower plasma intact PTH in healthy children and adults (38). In none of the studies, it was possible to show significantly higher levels of serum iFGF23 in subjects carrying the c.C716T variant.

Evaluation of phenotype in XLHR showed that growth was compromised, and there was a tendency for lower height *z*-scores in males than females. Also, we found a trend for males having a higher proportion of skeletal and dental manifestations than females. As discussed above, some studies points to a milder phenotype in females, with slight hypophosphatemia and mild or no overt skeletal disease (39, 40). There are also reports of slightly lower serum levels of phosphate (40, 41) and more severe skeletal disease in male than female XLHR patients (42). Other studies have reported no gender differences in skeletal phenotype (35, 43), but more severe dental phenotype in post pubertal males than females (35, 44). Thus, our findings support the notion of a more severe mineralization defect in males than females.

Dental involvement seemed to be less common in the patients who started treatment before 1 year of age, suggesting the importance of proper mineralization of dentin prior to the eruption of teeth (45). On the other hand, starting treatment before age 1 year did not lead to an improved height *z*-score at the last registered consultation. Some earlier studies have concluded that early start of treatment had a positive effect on linear growth (23, 46). In one study however, the height *z*-score was generally higher in those who started treatment before the age of 1 year compared with those who started later, but declined over time for those who started treatment early and improved in those who started treatment later (46). We found that treatment with phosphate and vitamin D improved mineral homeostasis and rickets, but did not fully correct skeletal axis deviation and to a lesser extent correct the growth deficiency in HR. This adds support to the theory that FGF23 may play a role in the normal physiology of mineralized tissues independently phosphate regulation (18). Treatment with phosphate will lead to transient increases in serum phosphate, which trigger production and release of FGF23 (47) and PTH (48), further aggravating the skeletal phenotype. Novel therapy with FGF23 neutralizing antibodies has shown that inhibition of excess FGF23 activity correct growth deficiency in mice (49), and anti-FGF23 antibodies are currently being tested in human XLHR (50, 51). It is possible that longitudinal growth in HH patients reflects the individual severity of and response to a disturbed FGF23 homeostasis, rather than the severity of hypophosphatemia itself.

The patients who developed nephrocalcinosis had started treatment earlier and had received higher daily doses of phosphate, but did not have better growth outcomes, than patients without nephrocalcinosis. Renal function remained normal in all patients, except for transient low-grade renal failure seen in the XLHR patient with tertiary HPT. Our results strengthen the association between higher phosphate doses and development of nephrocalcinosis found in earlier studies (52, 53, 54, 55). Early start of treatment as a risk factor for nephrocalcinosis has been found by some (52), but not by others (23, 46, 56). The prevalence of nephrocalcinosis in patients receiving combination therapy with phosphate and calcitriol is reported to be from 33 to 80% (median 59%) (23, 46, 52, 53, 54, 55, 56, 57, 58), but long term follow-up of mild nephrocalcinosis in XLHR does not seem to affect renal function (56). As discussed above, treatment with phosphate and calcitriol has a certain positive effect on growth, but only phosphate-treated patients develop nephrocalcinosis (54, 55, 56). This again probably reflects that current treatment options are suboptimal, both when considering skeletal outcome and the rate of complications.

Elevated serum levels of PTH were found in ten of 15 XLHR patients before the start of treatment all patients developed HPT during the course of treatment. Our findings add to other reports of high normal or slightly elevated levels of PTH in hypophosphatemic untreated XLHR patients (59, 60, 61). In normal subjects, hypophosphatemia will, through an increase in 1,25(OH)_2_D, reduce PTH levels (62). Evidence also suggests an inhibitory effect of FGF23 on PTH production (63). The explanation for the inappropriate PTH response in untreated HR, and the details of the interactions between phosphate, FGF23, and PTH, still need further clarification.

Secondary HPT caused by oral phosphate supplements can be counteracted by increasing the doses of calcitriol, with the risk of developing hypercalcuria and nephrocalcinosis, or by reducing the phosphate dose, with the risk of worsening rickets (64). However difficult, successful management of HPT in XLHR is important, as HPT has been associated with development of hypertension and renal failure (24, 65), cardiac failure (66), and also brown tumor in the mandible (67).

Two patients, one with XLHR, developed tertiary HPT after long-term use of phosphate supplements. The XLHR patient had received relatively high doses of phosphate and relatively low doses of alfacalcidol for more than 10 years. Tertiary HPT has been reported in 36 cases of HR (24, 65, 66, 68, 69, 70, 71, 72, 73, 74, 75), and prolonged treatment with high doses of phosphate supplements seems to be a risk factor (68, 71). There are reports of successful treatment of tertiary HPT with cinacalcet in children (24, 76) and adults (77, 78), but safety concerns have stopped further clinical trials investigating the effects of cinacalcet in children (79). A recent report suggests the vitamin D analog paricalcitol to suppress elevated PTH secondary to treatment in XLHR (80). However, careful monitoring of treatment, to ensure lowest efficient phosphate dose is very important to heal rickets and at the same time reduce the risk of tertiary HPT.

The observations from this study support recently published guidelines on treatment and monitoring of HR in children (64, 81). We recommend that combined treatment with oral phosphate and activated vitamin D (calcitriol or alfacalcidol) is started once the diagnosis has been made. Most children respond well to a calcitriol dose of 20–30 ng/kg per day (divided in two doses) or alfacalcidol 30–50 ng/kg per day (single dose) and an elemental phosphorous dose of 20–40 mg/kg per day (divided in four doses) with reduced signs of rickets and skeletal deformities. The starting doses of phosphate should be kept low to reduce gastrointestinal side effects, and to avoid complications clinical and biochemical controls should be performed at least every 3 months, and supplemented with skeletal X-rays every 2 years and renal ultrasonography every 2–5 years. To avoid HPT, the aim should not be normalization of serum phosphate, but the lowest efficient dose that promote growth and heal rickets. To minimize the risk of nephrocalcinosis, hypercalcuria, defined as U-Ca/creatinine ratio >0.87 mmol/mmol should be avoided.

One strength of our study is related to the fact that combined data from the NPR and all pediatric centers in Norway allowed us to collect a complete national material of childhood HH. This allowed for the estimation of a national prevalence, and adds information to the literature on the epidemiology of hereditary HR. Moreover, we have identified new mutations in known and novel genes, expanding the genetic diversity of HH with and without rickets. On the other hand, the study is limited by the size of the cohort and the retrospective design, implying we could not ensure uniform collection of information from the clinical, biochemical, and radiological examinations. Furthermore, we did not do genetic testing on normophosphatemic, asymptomatic siblings, as predictive genetic testing on children is not allowed in Norway according to the Biotechnology Act. This means there is a possibility for undiagnosed subclinical cases.

In conclusion, we have presented the first complete national cohort of HH in children. The prevalence of XLHR seems to be lower in Norwegian children than reported earlier.

## Supplementary data

This is linked to the online version of the paper at http://dx.doi.org/10.1530/EJE-15-0515.

## Author contribution statement

S Rafaelsen, H Ræder, S Johansson, and R Bjerknes designed the study; S Rafaelsen collected the data; whereas S Rafaelsen, H Ræder, S Johansson, and R Bjerknes contributed to data analysis and interpretation. S Rafaelsen and R Bjerknes drafted the manuscript, whereas all authors contributed to the revision and approved the final version of the manuscript.

## Figures and Tables

**Table 1:** Characteristics of the cohort of patients with hereditary hypophosphatemia^a^.

	**All patients** (*n*=28)	**XLHR** (*n*=21)
Time of diagnosis		
Sex (male/female) (*n*/*n*)	10/18	5/16
Family history of HH (*n*/*N*)	22/28	18/21
Age at diagnosis (years)	2.1 (0.1 to 15.5)	0.9 (0.1 to 15.5)
Height (*z*-score)	−0.9 (−6.5 to 1.0)	−1.2 (−6.5 to 1.0)
Skeletal disease^b^ (*n*/*N*)	17/28	13/21
Treatment		
Age at treatment start (years)	2.1 (0.2 to 15.6)	1 (0.2 to 6.7)
Elemental phosphorus (mg/kg per day)	39 (28 to 61)	39 (0 to 74)
Alfacalcidol (ng/kg per day)	33 (21 to 42)	34 (0 to 54)
Last registered consultation		
Age (years)	12.1 (1.3 to 18.3)	10.8 (1.3 to 18.0)
Height (*z*-score)	−1.4 (−6.31 to 0.8)	−1.4 (−6.3 to 0.8)
Delta *z*-score height (*z*-score)	−0.1 (−3.1 to 2.0)	−0.1 (−3.1 to 2.0)
Dental involvement (*n*/*N*)	13/28	9/21
Nephrocalcinosis (*n*/*N*)	11/28	9/20^c^
Persistent bowing (*n*/*N*)	16/28	13/21

*n*/*N*, number of patients with this characteristic/total number of patients.

a Continual variables are given as median (range).

b Skeletal disease: clinical or radiological signs of rickets, or skeletal axis deviation.

c Information missing for one patient.

**Table 2:** Effect of gender and early start of treatment in X-linked hypophosphatemic rickets^a^.

	**Stratified by gender**		**Age at treatment start**	
Male (*n*=5)	Female (*n*=16)	<1 year (*n*=10)	>1 year (*n*=9)
Time of diagnosis				
Age (years)	0.9 (0.5 to 15.5)	1.5 (0.1 to 6.5)	0.4 (0.1 to 0.9)	3.3 (0.7 to 15.5)
Height (*z*-score)	−3 (−5.1 to 0.5)	−0.9 (−6.5 to 1.0)	−0.8 (−3.0 to 1.0)	−2 (−6.5 to 0.5)
Skeletal disease^b^ (*n*/*N*)	4/5	9/15	3/10	9/9
Treatment data				
Age at treatment start (years)	1 (0.5 to 3.6)	1.1 (0.2 to 6.7)	0.6 (0.2 to 1.0)	3.6 (1.2 to 15.6)
Elemental phosphorus (mg/kg per day)	50 (32 to 64)	32 (0 to 74)	59 (11 to 74)	35 (28 to 67)
Alfacalcidol (ng/kg per day)	49 (37 to 54)	28 (0 to 48)	42 (17 to 54)	26 (17 to 37)
Last registered consultation				
Age (years)	14.8 (6.5 to 16.3)	7.9 (1.3 to 18.0)	11.1 (1.3 to 18.0)	8.4 (3.2 to 16.3)
Height (*z*-score)	−2.2 (−5.1 to −1.0)	−1.4 (−6.3 to 0.8)	−1.4 (−2.6 to 0.8)	−2 (−6.3 to 0.3)
Delta *z*-score (*z*-score)	0 (−2.1 to 1.3)	−0.2 (−3.1 to 2.0)	−0.4 (−3.1 to 2.0)	0 (−1.1 to 1.3)
Dental involvement (*n*/*N*)	4/5	5/15	2/10	7/9
Nephrocalcinosis (*n*/*N*)	2/5	7/15	7/10	2/9
Persistent bowing (*n*/*N*)	4/5	9/15	5/10	7/9

*n*/*N*, number of patients with this symptom/total number of patients.

a Continual variables are given as median (range).

b Skeletal disease: clinical or radiological signs of rickets, or skeletal axis deviation.
